# Accuracy of Mid-Upper Arm Circumference for Detecting Acute Malnutrition in Children Aged 6–59 Months in an Urban Slum in Bangladesh: A Cross-Sectional Analysis

**DOI:** 10.7759/cureus.33137

**Published:** 2022-12-30

**Authors:** Ahad Mahmud Khan, Robaiyat Sharmin, Md. Faizul Ahasan

**Affiliations:** 1 Public Health, Projahnmo Research Foundation, Dhaka, BGD; 2 Usher Institute, The University of Edinburgh, Edinburgh, GBR; 3 Department of Physiology, Dr Sirajul Islam Medical College, Dhaka, BGD; 4 Department of Pharmacology, Ibrahim Medical College, Dhaka, BGD

**Keywords:** children, weight-for-height, anthropometry, mid upper arm circumference(muac), acute malnutrition

## Abstract

Background

The weight-for-height z-score (WHZ) is considered the gold standard for detecting acute malnutrition in children. However, an accurate assessment of WHZ can often be challenging, especially in community settings. Mid-upper arm circumference (MUAC) is a simple and easy-to-perform method to identify children with acute malnutrition. The objective of the study was to evaluate the accuracy of MUAC in detecting acute malnutrition compared to WHZ among children aged 6-59 months in Bangladesh.

Methods

We used anthropometric data for 239 children aged 6-59 months from a cross-sectional study conducted in 2013 in an urban slum in Dhaka, Bangladesh. The sensitivity and specificity of MUAC to detect acute malnutrition were calculated, considering WHZ as the gold standard.

Results

The mean age of the children was 29.4 ± 12.7 months, with a male-to-female ratio of 1:1.2. The prevalence of acute malnutrition was 17.1% and 22.5% based on WHZ and MUAC, respectively. The total area under the receiver operating characteristic curve was 0.816. For detecting acute malnutrition, the sensitivity of MUAC was 61.0% and the specificity was 85.4% for the World Health Organization (WHO) recommended cutoff of <125 mm. Using the Youden index, the best MUAC cutoff point to detect acute malnutrition was <128 mm with a sensitivity of 75.6% and a specificity of 74.7%.

Conclusions

Our study demonstrated a low sensitivity of MUAC to identify acute malnutrition at the WHO cutoff of <125 mm. The cutoff could be increased to <128 mm to capture more children with acute malnutrition.

## Introduction

Acute malnutrition significantly increases the risk of childhood morbidity and mortality and is reported to be responsible for about 45 million global deaths in children younger than five years of age. More than two-thirds of these children live in Asia, and more than one-quarter live in Africa [1]. Children with acute malnutrition are affected by compromised immune systems, leaving them vulnerable to developmental delays, disease, and death [2].

The World Health Organization (WHO) recommends using the weight-for-height z-score (WHZ) as the gold standard to detect acute malnutrition. A child having a WHZ < −2 standard deviation (SD) is considered to be a case of acute malnutrition [3]. Accurate weight and height/length measurements and their conversion into WHZ can often be challenging.

Mid-upper arm circumference (MUAC) is a widely used method to define acute malnutrition in children aged 6-59 months. The WHO recommends a cutoff point of <125 mm to define acute malnutrition with MUAC [4]. MUAC is a simple, quick, and rapid method of detecting malnutrition, especially for mass screening and community-based diagnosis. There is a need for minimal supervision and training of health workers to detect acute malnutrition using MUAC [5].

The sensitivity and specificity of MUAC to diagnose acute malnutrition varied across the studies [6-10]. The prevalence of acute malnutrition in Bangladesh is among the highest in the world. About 8% of under-five children suffer from acute malnutrition in Bangladesh [11]. It is plausible that the accuracy of MUAC might be different in this setting, especially in a slum area where the prevalence of malnutrition is higher. In the present work, we evaluated the accuracy of MUAC in detecting acute malnutrition among children aged 6-59 months in an urban slum in Bangladesh.

## Materials and methods

This study used a cross-sectional dataset collected to investigate the association between maternal mental health and child nutritional status [12]. The study was undertaken from September to November 2013 in an urban slum area at Kamrangichar in Dhaka, Bangladesh. Kamrangichar has a surface area of 3.68 km2 and around 400,000 inhabitants. There were 12 administrative divisions, or "mohallas" in the study area. The present analysis included 239 children aged 6-59 months. An equal number of children were enrolled from each "mohalla." The data were collected by a team of two members. The data collectors started from the middle of each "mohalla," approached a random direction, visited consecutive households, and enrolled children.

A face-to-face interview with the mother was conducted to collect demographic and socioeconomic characteristics. Socioeconomic status was assessed using the modified Kuppuswamy's socioeconomic status scale [13]. Anthropometric measurements of the children were performed using standard techniques. The height/length was measured following the WHO guidelines [14]. For measuring weight, an electronic digital weighing scale was used. MUAC was measured with a non-stretchable and flexible MUAC tape manufactured by the United Nations Children's Fund (UNICEF) [15].

The anthropometric data were converted into WHZ using the software WHO Anthro [16]. The following cutoffs were used to define acute malnutrition: WHZ < −2 SD and MUAC < 125 mm. The accuracy of MUAC cutoffs was tested, considering WHZ as the reference measure. The sensitivity and specificity of MUAC were calculated using the WHO classification for acute malnutrition. To assess the accuracy of different MUAC cutoffs compared to WHZ < −2 SD, the receiver operating characteristic (ROC) curve was plotted, and the area under the curve (AUC) was calculated. To identify the MUAC cutoff with the optimum sensitivity and specificity, the Youden index was calculated [17]. The Statistical Package for Social Sciences (SPSS) software version 27 (IBM Corp., Armonk, NY, USA) was used to perform all analyses.

Ethical clearance was obtained from the National Institute of Preventive and Social Medicine (NIPSOM) in Bangladesh. The mothers provided informed written consent to participate in the study.

## Results

Table 1 shows the socio-demographic characteristics of the children. The majority of the respondents were in the age group of 24-35 months (34.7%), followed by 36-59 months (31.8%) and 12-23 months (28.5%), with a mean of 29.4 ± 12.7 months. The male-to-female ratio was 1:1.2. Most participants belonged to lower socioeconomic conditions (64.0%).

**Table 1:** Socioeconomic characteristics of the children

Variables	Number (%)
Age (months)	
6–11	12 (5.0)
12–23	68 (28.5)
24–35	83 (34.7)
36–59	76 (31.8)
Sex	
Male	108 (45.2)
Female	131 (54.8)
Socioeconomic status	
Lower	153 (64.0)
Lower middle	67 (28.0)
Upper middle	19 (7.9)
Total	239 (100)

The prevalence of acute malnutrition was 17.1% and 22.5% based on WHZ and MUAC, respectively (Table 2).

**Table 2:** Prevalence of acute malnutrition based on WHZ and MUAC WHZ: weight-for-height z-score, MUAC: mid-upper arm circumference

	Number (%)
WHZ	
Acute malnutrition (< −2 SD)	41 (17.1)
Normal (≥ −2 SD)	198 (82.8)
MUAC	
Acute malnutrition (<125 mm)	54 (22.5)
Normal (≥125 mm)	185 (77.4)
Total	239 (100)

**Figure 1:**
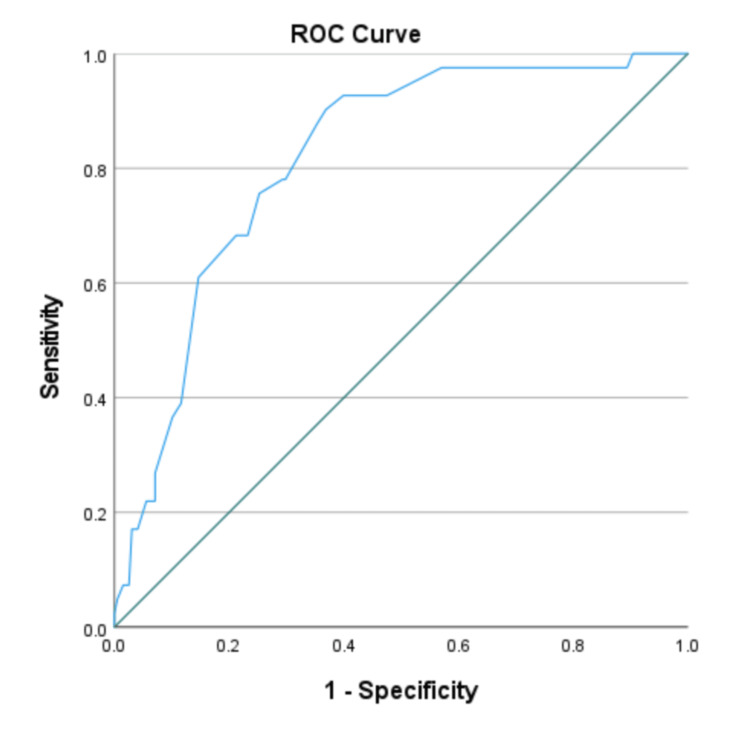
ROC curve plotting the sensitivity and specificity of MUAC for detecting acute malnutrition (WHZ < –2 SD as the gold standard) ROC: receiver operating characteristic

Figure 1 shows the ROC curve, and Table 3 summarizes the diagnostic test accuracies for varying MUAC cutoffs with WHZ < −2 SD as the gold standard. The AUC was 0.816 (95% CI 0.752-0.880). With <125 mm as the MUAC cutoff, 39.0% of children with acute malnutrition were missed. The optimal cutoff of MUAC was <128 mm, where the Youden Index was the maximum. The sensitivity at this cutoff of MUAC was 75.6%, and the specificity was 74.7%.

**Table 3:** Sensitivity, specificity, and Youden index at various cutoffs of MUAC for detecting acute malnutrition MUAC: mid-upper arm circumference

MUAC cutoff (mm)	Sensitivity (%)	Specificity (%)	Youden index (%)
<120	26.8	92.9	19.7
<121	36.6	89.9	26.5
<122	36.6	89.9	26.5
<123	39.0	88.4	27.4
<124	53.7	86.4	40.1
<125	61.0	85.4	46.4
<126	68.3	78.8	47.1
<127	68.3	76.8	45.1
<128	75.6	74.7	50.3
<129	78.0	70.7	48.7
<130	78.0	70.2	48.2

## Discussion

This study investigated the diagnostic accuracy of MUAC in comparison to WHZ to identify acute malnutrition in children aged 6-59 months in Bangladesh. The prevalence of acute malnutrition was 17.1% based on WHZ. The area under the ROC curve of 0.816 represents MUAC as a "very good" test for diagnosing acute malnutrition. There was a low sensitivity and a moderate specificity to the WHO-recommended MUAC cutoff of <125 mm. Various studies have reported low sensitivity of the current cutoff of MUAC [6,7,9]. Low sensitivity of MUAC may result in ineffective screening, and a significant proportion of children with acute malnutrition might remain unidentified.

This study proposed a new cutoff for MUAC to detect acute malnutrition in Bangladeshi children. The cutoff suggested based on the Youden index was <128 mm. The increase in MUAC cutoff by 3 mm increased sensitivity from 61.0% to 75.6% without much decline in specificity. Similar recommendations for increasing the MUAC cutoff for detecting acute malnutrition were also given in other studies [6,7,9,18]. A study conducted in Nepal reported the best cutoff for MUAC at <132 mm [7]. Another study conducted in India suggested <132 mm as the best MUAC cutoff [9]. A study in Bangladesh agreed with the WHO cutoff for children aged 6-24 months but proposed an increase to the cutoff of <135 cm for children aged 25-36 months and <140 cm for children aged 37-60 months [6]. The possible reason for the variability in cutoff points might be the difference in the body composition of the children in different settings. A higher revised cutoff will help to capture more children with acute malnutrition from the community. This might be beneficial for the early identification and appropriate management of acute malnutrition cases, and thus, the effective implementation of child nutritional programs.

According to the Bangladesh Demographic and Health Survey, the prevalence of acute malnutrition among under-five children is about 8% in Bangladesh [11]. However, the present study identified about 17.1% of children with acute malnutrition using WHZ. The possible reason for the higher prevalence of acute malnutrition is that the study was conducted in an urban slum area where most participants belonged to lower socioeconomic conditions. The proportion of acute malnutrition using the MUAC cutoff of <125 mm was 22.5%. However, the new cutoff of <128 mm identified about 33.9% of children as having acute malnutrition. The identification of a higher number of children with acute malnutrition may have an impact on the country's health system.

The strength of this study was that the validity of different MUAC cutoffs was tested in a community setting. A single team of data collectors was used to collect anthropometric data from all children to avoid inter-observer variation. Anthropometric measurements were performed using standard techniques. The UNICEF-recommended tape was used to measure MUAC.

The study had several limitations. First, WHZ was used as a reference standard, but WHZ itself is not without its challenges for the identification of acute malnutrition [19]. Second, both WHZ and MUAC show gender bias [20,21]. However, we could not perform any gender-specific analysis as the sample size for the study was relatively small. For the same reason, it was not possible to perform any age-specific analysis. There is poor agreement between MUAC and WHZ in older children [7,21,22]. Third, very few children were identified as cases of severe acute malnutrition (SAM). Therefore, we could not evaluate the diagnostic accuracy of MUAC in detecting SAM cases, and we restricted the analysis to the identification of acute malnutrition. Last, the participants were recruited from one selected urban slum in Bangladesh. Therefore, the results of the study may not reflect the exact picture of Bangladesh.

## Conclusions

The sensitivity of MUAC at the WHO-recommended cutoff is low for identifying acute malnutrition in children. The cutoff can be increased from <125 mm to <128 mm to increase sensitivity with a little compromise in specificity. The research was done based on available data from a particular area of Bangladesh. Hence, the study's results cannot be generalized. Further studies with a larger sample size and geographic representation can generate more evidence on the performance of MUAC in detecting acute malnutrition in children in Bangladeshi settings.
